# Impact of aerobic, resistance, and combined training on cardiometabolic health-related indicators in inactive middle-aged men with excess body weight and obesity

**DOI:** 10.3389/fphys.2025.1519180

**Published:** 2025-02-25

**Authors:** Friew Amare Mengistu, Yehualaw Alemu Lake, Molalign Enchalew Andualem, Yalemsew Demlie Miherete, Solomon Adamu Zewdie

**Affiliations:** Debre Markos University, Markos, Ethiopia

**Keywords:** fasting blood glucose, insulin resistance, resistance training, aerobic training, concurrent training

## Abstract

**Methods:**

Twenty physically inactive men (49.15 ± 2.581 years) and BMI with 27.66 ± 0.91, participated in an 8-month training programme involving concurrent exercise (CT), resistance training (RT), and aerobic training (AT) program to determine the effects on fasting blood glucose (FBG), insulin resistance (IR), blood pressure (BP) and waist–to–hip ratio (WHR) in overweight and obese adult persons. This study was used a randomized repeated measures parallel experimental design.

**Results:**

Pre-to-post mean values of FBG, IR, SBP, DBP and WHR significantly decreased. Exercise modality had a significant effect on FBG (F (2, 26) = 10.656, p = 0.001, η^2^ = 0.571), with RT and CT showing greater reductions than AT. IR decreased more in RT than in AT (MD = 0.410 ± 0.101, p = 0.03). SBP also varied significantly between modalities (F (2, 26) = 13.103, p = 0.02, η^2^ = 0.528), with CT and RT showing larger reductions than AT. WHR differed significantly (F (2, 16) = 18.175, p = 0.001, η^2^ = 0.694), with AT and CT showing more reductions than RT. Diastolic blood pressure (DBP) showed no significant effect from exercise modality.

**Conclusion:**

These findings highlight the importance of tailored exercise interventions, with short rest RT and CT emerging as the most effective method for inactive overweight and obese individuals.

## 1 Introduction

A significant public health concern is the increasing prevalence of adult obesity and related conditions. Obesity is a condition in which an abnormal or excessive accumulation of fat poses a health risk. The highest prevalence of obesity was 44.3% among middle-aged adults (40–59 years old) (Boutari and Mantzoros, 2022). The combined estimates of the prevalence of obesity and overweight in Middle Eastern countries are 21.17% and 33.14%, respectively (Okati-Aliabad et al., 2022). Like in many other countries, overweight and obesity are becoming increasingly prevalent public health issues in Ethiopia (Kassie et al., 2020). It is important to prioritize strategies that can help reduce the healthcare costs associated with obesity. Researchers strongly suggest that interventions improving both blood sugar control and cholesterol levels would be highly effective in preventing cardiovascular diseases (De Backer et al., 2003).

As highlighted in the 2025 ACSM Worldwide Fitness Trends, the Exercise is Medicine (EIM) initiative emphasizes incorporating physical activity into routine healthcare practices. Since its debut on the trends list in 2017, EIM has consistently ranked among the top 20 trends, reflecting its enduring relevance and impact (Newsome et al., 2024). Despite progress in understanding how different forms of exercise can mitigate obesity and related health risks, there remains significant debate and uncertainty about the optimal activity levels required for maximum benefits. Aerobic exercise, for example, predominantly utilizes fat as an energy source, enhancing the body’s ability to break down stored fat through lipolysis (Brown, 2002; Noland, 2015). This process not only reduces fat stores but also has a cascade of beneficial effects on metabolic health. Enhanced lipolysis reduces ectopic fat accumulation in organs like the liver and muscle, which is a key driver of insulin resistance. As a result, insulin sensitivity improves, leading to better regulation of blood glucose levels (Bruno et al., 2018). Additionally, the reduction in fat mass also alleviates strain on the cardiovascular system, aiding in the normalization of blood pressure. This occurs through mechanisms such as improved endothelial function, reduced arterial stiffness, and enhanced nitric oxide production, which collectively support better vascular health and circulation.

High-intensity strength training burns mostly carbohydrates for immediate energy (Brown, 2002). It also triggers the release of hormones such as growth hormone and testosterone (Kraemer et al., 2020). By influencing the body’s chemistry, hormones promote muscle growth and make it easier to access the body’s ability to burn glucose derived from fat stores (gluconeogenesis) (Coll-Risco et al., 2016; Loucks and Caiozzo, 2012). To assess the impact of exercise programs on health accurately, participants need to follow a strict dietary monitoring protocol (Matthews et al., 2012) to ensure that any observed changes are primarily due to the exercise intervention itself and not influenced by significant shifts in their dietary habits (Beck et al., 2015).

Researchers have previously studied the separate effects of aerobic exercise and strength training on health (Doewes et al., 2023; Kim et al., 2019; Mann et al., 2014). While some research shows that these exercises combined can help older adults with obesity manage their blood sugar and cholesterol (Azarbayjani et al., 2014; Batrakoulis et al., 2022), blood pressure and insulin resistance (Cornelissen et al., 2011; Kolahdouzi et al., 2019), a knowledge gap exists in comparing aerobic, resistance, and concurrent training, particularly in studies that control for participants’ dietary practices. We lack a clear understanding of how these training modalities differ under controlled dietary conditions.

Hence, the objective of this study was to assess and compare the efficacy of various exercise modes (aerobic, resistance training, and combined exercise) and their ability to increase metabolic biomarkers, pressure and anthropometric indices over time among adults with overweight and obesity.

## 2 Methods and materials

This study is reported following the CONSORT guidelines.

### 2.1 Research setting and design

This study used a randomized parallel group experimental design to evaluate between-group differences to track between-subject difference after 12-week training.

Males aged 45–60 years who were physically sedentary and had a BMI greater than 24.9 kg/m2 were included in the study. The volunteers were chosen from among the inactive citizens of Debre Markos town, Ethiopia, and were notified via notice boards and local radio.

The inclusion criteria were as follows: (a) had a BMI >24.9 kg/m^2,^ (b) were aged between 45 and 60 years, (c) volunteered to participate, (d) were physically inactive (did not achieve 30–60 min per day or 150 min per week of moderate intensity exercise or 20–60 min per day (75 min per week) of vigorous intensity (Cohen, 1991) and cleared a medical history from the physical activity readiness questionnaire), and (d) were able to perform the necessary exercises. The exclusion criteria were as follows: (a) any cardiovascular, respiratory, or musculoskeletal disorders precluding physical exercise; (b) uncontrolled hyperglycaemia (≥126 mg/d) or hypertension (a resting blood pressure ≥140/100 mmHg); and (c) active infection, (d) acute myocardial infarction, stroke, trauma, surgery or severe liver dysfunction.

Using the G*Power tool, we were able to identify the optimal sample size for our experiment. In accordance with Sousa et al. (2014), we calculated a sample size of 21 individuals, accounting for zero correlations between AT TGC, total-c, LDL-c, and HDL-c, a significance threshold (α) of 0.05, lower effect size 0.25, with 15 number of measurements and a power of 0.95 (Sousa et al., 2014). Considering a 10% non response rate, a total sample size of 24 was needed. By successfully detecting the specified effect size with this sample size, we were able to reach the desired statistical power and significance level for our investigation.

**FIGURE 1:**
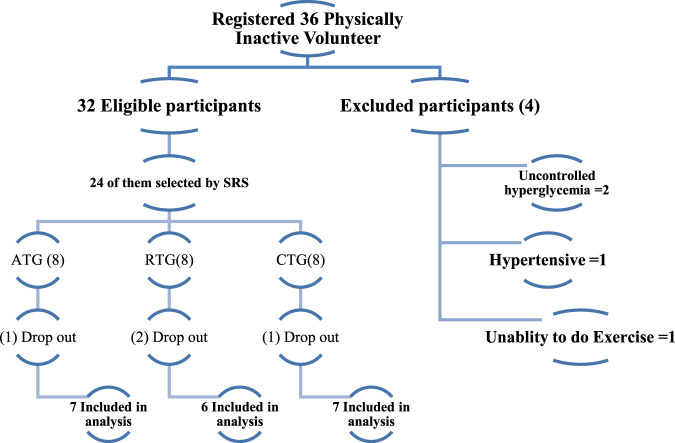
Participant description chart.

Thirty-six men who were overweight or obese were recruited and registered for the current study. Of the 36 volunteers we had originally registered as physically inactive, 32 remained when the inclusion criteria were applied. Using a straightforward random selection process, the researchers produced a final study group of 24 volunteers. Those who participated were then randomly assigned to one of three exercise groups, each consisting of eight participants: aerobic, resistance, or combination training. Balanced randomization (1:1:1) and homogeneous samples were used in the investigation (Figure 1). Throughout the investigation, the data collectors were blinded to one another.

To ensure ethical conduct, participants were fully informed about all procedures, risks, and guidelines before providing their informed consent. According to Cohen, 1991, this met the standards established by the American College of Sports Medicine (Cohen, 1991). Additionally, all procedures involving human subjects were examined and given feedback by Debre Markos University Sport Science Academy Ethics Review Committee (ERC) (SPSC05/22). Finally, the ethical guidelines outlined in the 2000 amendment of the Declaration of Helsinki were followed when the study was conducted. This trial was retrospectively registered on the Open Science Framework (OSF) platform. The registration can be accessed at https://doi.org/10.17605/OSF.IO/34MKQ.

The first set of data was gathered before the intervention began, and the second set was gathered at the completion of the 8-week intervention. The Debre Markos Referral Hospital is where the data were gathered. Systolic blood pressure (SBP), diastolic blood pressure (DBP), and the waist‒to-hip ratio were secondary objectives, whereas fasting blood glucose (FBG) and insulin resistance (IR) were the primary outcomes. The follow-up (intervention) periods spanned April 20/2022–2022.

### 2.2 Measurement of study variables

#### 2.2.1 Fasting glucose and insulin resistance

To minimize acute exercise effects and ensure accurate results, blood samples were collected 48 h post training. The participants fasted for 12 h and abstained from alcohol, coffee, and high-fat meals prior to testing. Blood samples were drawn by a trained medical laboratory technician at Debre Markos Referral Hospital via antecubital vein puncture, both before and after 12 weeks of exercise training, with a total volume of 5 mL collected.

The samples were centrifuged to separate the plasma from the cellular components and were stored at −80°C to maintain integrity for analysis. Glucose levels were measured via the hexokinase method (COBAS, Roche), with an intra coefficient of variation between 1.58% (µ = 64.7 mg/dL) and 1.38% (µ = 369 mg/dL) (Landberg et al., 2021). Fasting plasma insulin was quantified via a Human Insulin ELISA Kit (Shanghai Kehua Bio-Engineering Co., Ltd., China) (Shakil-Ur-Rehman et al., 2017).

The homeostasis model of insulin resistance (HOMA-IR index), which is the product of glucose and insulin concentrations divided by a factor, was used to quantify insulin resistance (Vogeser et al., 2007). The HOMA-IR index was calculated as follows:

$$HOMA-IR=\frac{\text{fasting}\;\text{serum}\;\text{glucose}\;\left(\text{mmol}.L^{-1}\right)*\text{fasting}\;\text{serum}\;\text{insulin}\left(\mu U.{mL}^{-1}\right)}{22.5}$$

#### 2.2.2 Blood pressure

Blood pressure was measured via an automated Sphygmocor XCEL device (AtCor Medical, CardieX, Sydney, Australia) (De la Torre Hernández et al., 2021). The appropriately sized cuff was placed on the participant’s left upper arm while they lay still on the catheterization laboratory table. After standard Oscillometric brachial blood pressure was recorded, the cuff was reinflated to sub diastolic pressure to capture volumetric waveforms for 5 s. The participants were instructed to keep their arms relaxed and refrain from movement during the measurement.

#### 2.2.3 Waist–hip ratio (WHR)

Waist and hip circumferences were measured via a Sammons Preston Tape (Narang Medical Limited, New Delhi) (Khan et al., 2009), with measurements taken to the nearest 0.1 cm. When waist circumference was measured, the participants were instructed to breathe normally and wear lightweight clothing to ensure accurate measurement. The measurement was taken at the narrowest point of the waist, usually located just above the navel. For hip circumference, measurements were taken at the site of the greatest circumference around the buttocks (Hu et al., 2010). The waist‒to-hip ratio was calculated as the waist circumference divided by the hip circumference (Bredella et al., 2009). All measurements were conducted by a trained data collector who has received comprehensive instruction in these measurement procedures. This training ensures that the data collector adheres to standardized protocols, promoting accuracy and consistency in the measurement process.

#### 2.2.4 Average daily energy intake

We utilized a 24-hour interactive questionnaire with several passes that was developed and validated for use in developing countries (Gibson and Ferguson, 1999). The three 24-hour sessions were held on Monday, Wednesday and Saturday to capture variation in intake throughout different days of the week. We applied the Ethiopian food composition table to estimate nutrient and energy levels from dietary data. The names of foods and drinks, their descriptions, cooking methods, and amounts from both 24-hour periods were coded and submitted to the NutriSurvey200 (Regassa et al., 2021). After the frequency of consumption per day was determined, we used the product sum approach to determine daily food intake. Daily food intake = ∑ (food item’s stated consumption frequency, translated to times per day) * (portion size ingested of that food). The daily average energy intake was also determined as follows: ADEi = ∑daily food intake/number of data collection days.

### 2.3 Exercise intervention protocol

Over the course of 8 weeks, the exercise routines consisted of three weekly sessions, each lasting 60 min. The participants warmed for five to 10 minutes before beginning 30–40 min of main training for each exercise session. They finished with a cool-down that lasted five to 10 min. The six exercises in the RT program were designed to target the body’s major muscle groups. Standing plantar flexion, squatting, machine leg pressing, neutral reowing, dumbbell curl, triceps pulley, bicep curl, and vertical bench pressing were the workouts that were performed. Three sets of eight to twelve repetitions at an intensity between fifty and seventy-five percent of their one-repetition maximum (1RM) were part of the training regimen. The remaining time between training sessions and between sets was approximately 48–72 h and 1–1.5 min, respectively (Jambassi Filho et al., 2020).

To determine protocol loads, the 1-RM test will be applied by gradually increasing resistance until the volunteer succeeded in performing no more than one repetition. Start with a warm-up that includes a small weight that is roughly 40%–60% of the perceived maximum load in order to calculate each individual’s 1-RM. To make sure the muscles are prepared for the subsequent section of the test, give everyone a minute to relax after finishing the warm-up. provide the individual 12–15 repetitions after increasing the weight to a moderate load (60%–80% of the perceived maximum). This set should be difficult, but not impossible. Rest for one to 2 minutes after finishing this set. After establishing a reasonable weight, encourage the subject to do up to 10 repetitions with a 10% increase in load. Allow the individual an additional one to 2 min of break before continuing if they are able to perform ten or more repetitions with the new weight. Keep an eye on the participant throughout this rest period to make sure they aren't exerting themselves excessively. Increase the weight by 10% again and have them try repetition with the larger load if they are able to finish the set of 10 or more reps. After that, the resistance will be gradually raised until the individuals could only complete each exercise nine repetition or less. Reaching the target number of repetitions in between 3 and 6 tries is the aim of increasing the resistance. Three minutes of rest are permit in between each particular exercise, and 2 min will be permitted between each try. Brzycki 1-RM prediction equation (Brzycki, 1993) will then use to estimate the 1-RM based on the resistance and repetitions recorded on the last try. The mathematical expression for the equation is 1RM = W/[102.78–2.78(R)]/100, where R is the maximum number of repetitions and W is the weight was using (Abdul-Hameed et al., 2012).

A treadmill was used for aerobic activity, and the intensity level ranged from 50% to 75% of the maximal heart rate (HRmax). Following a training regimen consisting of three exercises per session, the CTs completed the same number of workouts as the ATs and RTs did.

We will use the heart rate reserve (HRR) approach, which is based on the Karvonen formula, to determine the target heart rate (THR) in order to manage the intensity of the exercise (Yabe et al., 2021). This approach is appropriate for a broad spectrum of adult fitness levels (ACSM’s, 2013). The following is the formula:

$$\text{THR}=\text{HR rest}+\left[\left(\text{HR }\max-\text{HR rest}\right)\times \text{Intensity}\right]$$

Participants wore Polar heart rate monitors (Polar Electro, Kempele, Finland) to track their heart rate (Hernández-Vicente et al., 2021), ensuring that aerobic exercise was conducted within the desired percentage of their maximum heart rate (HRmax). These approaches ensured that participants performed the exercises at the prescribed intensity levels safely and effectively.

In each session, participants performed endurance exercises before moving on to strength exercises. Details of the general training intervention approach are outlined in Table 1.

**TABLE 1:** Training protocol details.

Weeks	Intensity				Duration			
	AT (HR _max_)	RT (1RM)	CT		AT (minutes)	RT (3 sets)	CT	
			AT	RT			AT	RT
Week 1 and 2	50%–55%	50%–55%	50%–55%	50%–55%	25	10–12	13	10–12
Week 3 and 4	55%–60%	55%–60%	55%–60%	55%–60%	30	10–12	15	10–12
Week 5 and 6	60%–65%	60%–65%	60%–65%	60%–65%	35	10–12	17	10–12
Week 7 and 8	65%–75%	70%–75%	65%–75%	65%–75%	40	10–12	20	10–12

This specific exercise order was selected to explore the impact of aerobic training preceding strength training (Lepers et al., 2001). All interventions were conducted under the supervision of qualified professionals to ensure adherence to the protocol, monitor participant safety, and provide guidance during the exercise sessions. And to minimize potential confounding factors, participants were explicitly advised not to engage in any additional resistance-type or aerobic training throughout the duration of the study.

### 2.4 Statistical analysis

SPSS version 26 was used to analyse the data (SPSS Inc., Chicago, IL). By using a Bonferroni adjustment for multiple comparisons in their ANCOVA, with average daily energy intake as the covariate, the researchers were able to secure dependable results for FBG, IR, SBP, DBP, and WHR. The various exercise types performed by the groups were compared, with statistical significance set at a p-value of 0.05 or lower. All statistical analyses were conducted using two-tailed tests.

## 3 Results

The baseline descriptive data characteristics and the adjusted absolute changes in FBG, IR, SBP, DBP, and WHR levels over the course of the study are summarized in Table 2. Twenty people completed the study and were analysed: seven in the AT, six in the RT, and seven in the CT. Four participants were dropped from the experiment due to exercise-induced injuries, parting a total of 20 participants who finished the study and were included in the analysis, 7 in the AT, 6 in the RT and 7 in the CT. The normality of the data was assessed using the Shapiro-Wilk test. The results indicated that all variables were normally distributed at baseline and follow-up (p > 0.05).

**TABLE 2:** Baseline and follow-up data while adjusting dietary practice.

	ATG (Age = 49.00 ± 2.08, ADEi = 2885.00 ± 109.180)		RTG (Age = 49.83 ± 3.06) ADEi = 2636.00 ± 98.82)		CTG (Age = 48.71 ± 2.87) ADEi = 2753.70 ± 178.12)	
	Baseline	Follow-up	Baseline	Follow-up	Baseline	Follow-up
FBG	98.49	94.44^a^	98.70	86.07^a^	96.83	88.61^a^
IR	2.822	2.17^a^	2.793	1.76^a^	2.820	1.963^a^
SBP	132.14	125.13^a^	130.50	123.72^a^	131.42	120.67^a^
DBP	87.14	87.147^a^	85.83	86.064^a^	84.00	83.798^a^
WHR	1.17	0.934^a^	1.21	1.074^a^	1.15	0.868^a^

Note. FBG: fastening blood glucose; IR: insulin resistance; SBP: systolic blood pressure; DBP: diastolic blood pressure and WHR: waist to hip ratio. The values are presented as the means.

^a^ Covariates appearing in the model were evaluated at average daily energy intake (ADEi) = 2671.5620_a_.

The results revealed no significant differences in any of the variables among the three groups in the pretest, suggesting successful randomization of the study participants. The average ages of the participants in the respective groups were AT = 49.00 ± 2.08, RT = 49.83 ± 3.06 and CT = 48.71 ± 2.87. The average body mass indices of the participants in the AT, RT and CT groups were 27.84 ± 1.10, 27.45 ± 0.74 and 27.70 ± 0.85, respectively (Table 2).

As Table 3 shows, exercise modality had a significant main effect on FBG (F (2, 26) = 10.656, P = 0.001, η^2^ = 0.571), as shown in Table 3. Post hoc analyses using the Bonferroni *post hoc* criterion revealed that RT (−8.376 ± 2.032, p = 0.002) and CT (MD = −5.837 ± 1.936, P = 0.025) were significantly greater than AT was. The IR from the HOMA-IR revealed a more substantial decrease in RT than AT did (MD = 0.410 ± 0.101, p = 0.03); nonetheless, there was no noticeable shift from the concurrent training group.

**TABLE 3:** Test between subject effect changes in outcomes within treatment groups.

Variables	Between-subjects effects			Pairwise comparison					
	F	Sig.^b^	η^2^	Treatment groups	Mean difference	Std. Error	Sig.^b^	95% CID	
								Lower bound	Upper bound
FBG	10.651	0.001	0.571	AT-RT	8.376	2.032	0.002	2.945	13.808
				AT-CT	5.837	1.936	0.025	0.661	11.012
				RT-CT	−2.540	2.444	NS	−9.073	3.993
IR	8.957	0.002	0.528	AT-RT	0.410	0.101	0.003	0.139	0.680
				AT-CT	0.211	0.096	NS	−0.047	0.469
				RT-CT	−0.199	0.122	NS	−0.524	0.127
SBP	6.33	0.009	0.442	AT-RT	1.415	0.915	NS	−1.032	3.862
				AT-CT	4.457	0.872	0.000	2.125	6.789
				RT-CT	3.042	1.101	0.042	0.099	5.986
DBP	4.849	0.023	0.377	AT-RT	1.084	2.005	NS	−4.276	6.443
				AT-CT	3.349	1.910	NS	−1.758	8.456
				RT-CT	2.265	2.412	NS	−4.181	8.712
WHR	16.175	0.001	0.594	AT-RT	−0.140	0.029	0.001	−0.218	−0.062
				AT-CT	0.066	0.028	NS	−0.008	0.140
				RT-CT	0.206	0.035	0.000	0.112	0.300

Note: FBG: fastening blood glucose; IR: insulin resistance; SBP: systolic blood pressure; DBP: diastolic blood pressure and WHR: waist to hip ratio; NS: not significant. Covariates appearing in the model are evaluated at the following values: average daily energy intake = 2671.5620.

Systolic blood pressure parameters F (2, 26) = 6.33, p = 0.009, and η^2^ = 0.442 were substantially lower in the CT groups than in the AT and RT groups (MD = 4.457, SE = 0.782, P ˂ 0.001) and (MD = 3.042, SE = 0.101, P ˂ 0.042), respectively. The waist-to-hip ratio varied significantly between the training modalities (F (2, 16) = 18.175, P = 0.001, η^2^ = 0.694). Both AT and concurrent training significantly decreased compared with RT (MD = 0.140, SE = 0.029, P = 0.01) and (MD = 0.206, SE = 0.035, P ˂ 0.001), respectively. Even though the diastolic blood pressure data were significantly different between the pretest and posttest values, the independent variables had no discernible effect on these variables.

## 4 Discussion

The current study revealed significant reductions in FBG across all groups, with the most substantial decreases observed in the short rest resistance training (RT) and concurrent training (CT) groups. Studies have shown that both concurrent (Al-Mhanna et al., 2024; Al-Mhanna et al., 2023; Castaneda et al., 2002; Sigal et al., 2007) and resistance training (Castaneda et al., 2002; Fink et al., 2018) improve glycemic control in adults with overweight and obesity. However, Cai and Zou (2016) conducted a meta-analysis of randomized controlled trials and reported that regular aerobic exercise can help manage FBG levels (Cai and Zou, 2016). Our research, however, casts doubt on this idea by showing that short rest RT can dramatically reduce FBG, suggesting that resistance training could be essential for glycemic control. This result was supported by Baldi and Snowling (2003) and Marquis-Gravel et al. (2015). In contrast, research on sprint interval training (SIT) by Ghaedi et al. (2020) did not supports this outcome.

The effectiveness of exercise in improving insulin sensitivity was highlighted by the significant decreases in IR observed in all groups, especially in short rest RT, which supported various findings (Mavros et al., 2013; Van Der Heijden et al., 2010; Yaspelkis, 2006) and also improves in SIT training (Ghaedi et al., 2020). Conversely, de Matos et al. (2018) reported that obese individuals engaging in high-intensity interval training showed no improvement in insulin resistance. In contrast to our findings, however, studies have shown that regular aerobic exercise (Bruno et al., 2018; Motahari-Tabari et al., 2014) and concurrent training (Bharath et al., 2018; Medeiros et al., 2015) can improve insulin resistance in obese individuals. This discrepancy, which may result from differences in study design, exercise order in concurrent training or intervention duration, emphasizes the potential advantages of including resistance training in exercise programs for the treatment of IR.

The observed decreases in SBP were most pronounced in the CT and RT groups, supporting findings from Lemes and his teammates (2016), which suggest that resistance training is effective in lowering systolic blood pressure in metabolic syndrome patients (Lemes et al., 2016). Another study demonstrated that concurrent training decreased systolic blood pressure in hypertensive individuals (Aguiar et al., 2017; Caminiti et al., 2022). Conversely HIIT training did not demonstrate significant improvement in resting blood pressure (Dunstan et al., 2002). Dimeo et al. (2012), on the other hand, noted that aerobic training significantly decreased systolic and diastolic daytime ambulatory blood pressure (Dimeo et al., 2012). Our findings support a dual-modality exercise strategy by confirming that resistance training and combination training offer greater advantages for reducing SBP.

Although all the groups showed significant reductions in DBP (Wewege et al., 2018), no substantial differences emerged between the training modalities (Moeini et al., 2015) except for the control group (Ambelu and Teferi, 2023). This finding is in line with that of Cornelissen and Smart (2013), who noted that diastolic pressure may be influenced by a variety of factors, including baseline health and diet. Conversely, a study reported that 9 months of aerobic exercise and concurrent training exercise can differentially affect diastolic blood pressure (Sousa et al., 2013), indicating that the length of training may influence how these interventions affect DBP.

Significant improvements in WHR were observed, particularly in the CT and aerobic training groups. This result supports previous findings that aerobic exercise (Chiu et al., 2017; Marandi et al., 2013) and concurrent exercise positively influence WHR and metabolic health (Ho et al., 2012; Monteiro et al., 2015). However, according to Ramos-Campo et al. (2021), resistance exercise can effectively reduce body composition (Ramos-Campo et al., 2021). Our results challenge this by demonstrating substantial reductions in WHR across both aerobic and combined training modalities, emphasizing the importance of various exercise approaches in managing body composition. Overall, the findings of this study reinforce the significant role of tailored exercise interventions in improving key metabolic and cardiovascular health markers in overweight and obese adults. The contrasting results with the literature highlight the complexities of exercise effects and underscore the need for further research to elucidate the optimal exercise strategies for different populations.

One limitation of this study is the absence of a non-intervention control group, which restricts the ability to fully attribute observed changes solely to the intervention. While the study was designed with random assignment and control of key confounding factors, the lack of a traditional control group limits the ability to isolate the effects of the intervention from other potential influences. Future research should consider incorporating a non-exercising control group to strengthen the validity of causal inferences and provide a more comprehensive understanding of the intervention’s impact. However, the observed differences in the effectiveness of each modality suggest that individualized exercise prescriptions could optimize outcomes, especially for older adults. Future studies should explore the long-term effects of these interventions and evaluate whether combining different modalities yields synergistic benefits. Additionally, more diverse participant groups and the inclusion of non-exercising control groups will be essential in validating these results and refining clinical guidelines.

## 5 Conclusion

The results of the current study confirm the undeniable benefits of regular exercise for overweight and obese adults. Specifically, concurrent training (CT), which combines short rest resistance training and continuous aerobic training, proves to be the most effective approach for improving metabolic and cardiovascular health indices, as well as enhancing the waist-to-hip ratio in overweight and obese individuals. These findings highlight the importance of incorporating CT as a primary exercise strategy for improving overall health and managing weight-related conditions.

## Data Availability

The datasets generated and/or analyzed during the current study are available from the corresponding author upon reasonable request.
